# A Surgical Handbook, for the Use of Students, Practitioners, House-Surgeons, and Dressers

**Published:** 1889-12

**Authors:** 


*A Surgical Handbook, for the use of Students, Practitioners, House-Surgeons, and Dressers.* By F. M. CAIRD, M.B., F.R.C.S.; and C. W. CATHCART, M.B., F.R.C.S. Pp. 262. London: C. GRIFFIN and Co. 1889.

This is the best handbook for surgical dressers and house-surgeons with which we are acquainted. Compact in language and in bulk, very complete in matter, and thoroughly up to date, it is a pleasing contrast to the well-known, oldfashioned, and much re-edited books on bandaging with which our youth was familiar. Besides the ordinary information as to bandaging and dressing and the application of splints, there are very excellent chapters on anæsthetics, the treatment of emergencies, the surgical applications of electricity, the examination of the urine, the taking of plaster casts, and a host of minor matters. Small type and fine paper have rendered it possible to give a surprisingly large amount of information in a very small book compass. The elegances of gilt edges and leather covers becomingly set off a work which might worthily be the constant companion of every surgical student in the country.

